# *In vivo* inhibition of angiogenesis by sulphamoylated derivatives of 2-methoxyoestradiol

**DOI:** 10.1038/sj.bjc.6603727

**Published:** 2007-04-10

**Authors:** S K Chander, P A Foster, M P Leese, S P Newman, B V L Potter, A Purohit, M J Reed

**Affiliations:** 1 Endocrinology and Metabolic Medicine and Sterix Ltd, Imperial College, St Mary's Hospital, London W2 1NY, UK; 2 Medicinal Chemistry and Sterix Ltd, Department of Pharmacy and Pharmacology, University of Bath, Bath BA2 7AY, UK

**Keywords:** breast cancer, angiogenesis inhibitors, 2-methoxyoestradiol, sulphamates, Matrigel plug assay

## Abstract

Drugs that inhibit growth of tumours and their blood supply could have considerable therapeutic potential. 2-Methoxyoestradiol-3,17-*O,O*-*bis*-sulphamate (2-MeOE2*bis*MATE) has been shown to inhibit the proliferation of MCF-7 (ER+) breast cancer cells and angiogenesis *in vitro*. 2-MeOE2*bis*MATE and its analogue, 17-Cym-2-MeOE2MATE, were investigated for their ability to inhibit *in vivo* angiogenesis and tumour growth. The mouse Matrigel plug assay for angiogenesis was used to investigate the effect of compounds on neovascularisation and was quantified using a FITC-dextran injection technique. Nude mice bearing tumours derived from MCF-7 cells were used to assess efficacy on tumour growth. Tumour sections were stained for VEGFR-2 and Ki67 to assess tumour angiogenesis and cell proliferation respectively. Matrigel plugs supplemented with basic fibroblast growth factor resulted in increased neovascularisation over 7 days. Oral administration of 2-MeOE2*bis*MATE for 7 days at 10 or 50 mg kg^−1^ significantly reduced neovascularisation to or below control levels respectively. 17-Cym-2-MeOE2MATE at 20 mg kg^−1^ was equally effective. 2-MeOE2*bis*MATE, dosed daily for 21 days, caused a 52% reduction in tumour growth at 5 mg kg^−1^ and 38% regression at 20 mg kg^−1^. 17-Cym-2-MeOE2MATE (20 mg kg^−1^) reduced tumour growth by 92%. Immunohistochemistry revealed a reduction in angiogenesis and proliferation. Matrigel plug and tumour imaging after FITC-dextran injection indicated that 2-MeOE2*bis*MATE caused a marked disruption of vasculature. These sulphamoylated oestrogen derivatives have been shown to be potent inhibitors of angiogenesis *in vivo.* This, together with their ability to inhibit tumour growth, indicates the potential of this new class of drugs for further development for cancer therapy.

The development of angiogenesis inhibitors, which block the formation of new blood vessels, offers a novel therapeutic approach to inhibit tumour growth (Folkman, 1992). A number of anti-angiogenic drugs have now been developed and tested in clinical trials (Harris, 1997; Zhang and Harris, 1998). So far, the use of anti-angiogenic agents to inhibit tumour growth has met with only limited success. In a recent study, a monoclonal antibody against vascular endothelial growth factor (VEGF), bevacizumab, in combination with chemotherapy resulted in an improvement in survival in patients with colorectal cancer (Hurwitz *et al*, 2004). This suggests that to obtain the best results with anti-angiogenic drugs they will need to be used in conjunction with cytotoxic agents. Support for this concept has emerged from studies in which chemotherapeutic agents are administered in a metronomic low-dose schedule in an attempt to combine their anti-proliferative and anti-angiogenic activities (Vacca *et al*, 1999; Hanahan *et al*, 2000). Over the last decade there has been considerable interest in the natural oestrogen metabolite, 2-methoxyoestradiol (2-MeOE2, Figure 1, **1**) as a potential drug for cancer therapy (Seegers *et al*, 1989; Fotsis *etal*, 1994; Klauber *etal*, 1997; Brem, 1998; Zhu and Conney, 1998a, 1998b; Lakhani *et al*, 2003; Dahut *et al*, 2006). This compound not only inhibits the proliferation of cancer cells *in vitro* and tumours *in vivo* but also displays anti-angiogenic activity (Fotsis *etal*, 1994; Klauber *et al*, 1997). 2-MeOE2 is currently in phase I/II trials for the treatment of breast and prostate cancer but relatively high doses (up to 6 g day^−1^) are used (Dahut *et al*, 2006). The reason for this is that 2-MeOE2 has a very low bioavailability and is rapidly inactivated by conjugation and oxidation of the hydroxyl groups at the C3/C17 positions of the oestrane nucleus (Liu *et al*, 2005; Newman *et al*, 2006). Furthermore, 2MeOE2 has been shown to exhibit mitogenic effects in ER-positive cells that were mediated through the oestrogen receptor (Liu and Zhu, 2004). Numerous analogues of 2-MeOE2 have been synthesised and tested in an attempt to improve its potency including the 2-ethoxy, 2-methoxymethyl and 14-dehydro derivatives (Cushman *et al*, 1995; Brueggemeier *et al*, 2001; Tinley *et al*, 2003). However, all these analogues retain the C3/C17 hydroxy groups of 2-MeOE2 and it is likely that they, like 2-MeOE2, will be rapidly inactivated *in vivo*.

As an alternative approach to modification of either the steroid nucleus or the C2 position of 2-MeOE2, the C3/C17 hydroxy groups were sulphamoylated to give 2-methoxyoestradiol-3,17-*O*,*O*-*bis*-sulphamate (2-MeOE2*bis*MATE, Figures 1, 2) (Howarth *et al*, 1994; Purohit *et al*, 1995a, 1995b; Raobaikady *et al*, 2003; Newman *et al*, 2004; Leese *et al*, 2006). In addition, a C17 analogue of 2-MeOE2*bis*MATE, cyanomethyl derivative (2-methoxy-3-*O*-sulphamoyl-17*β*-cyanomethyloestra-1,3,5(10)-triene,17-Cym-2-MeOE2- MATE, Figures 1, 3) was also synthesised (Utsumi *et al*, 2005). 2-MeOE2*bis*MATE was previously shown to inhibit angiogenesis in two *in vitro* assays (Newman *et al*, 2004). It inhibited the proliferation of human umbilical vein endothelial cells (HUVECs), used as a marker of angiogenesis, with the *bis*-sulphamate being 60-fold more potent than 2-MeOE2. In addition, using an endothelial cell/fibroblast co-culture model of *in vitro* angiogenesis the *bis*-sulphamoylated derivative was 13-fold more potent at inhibiting tubule formation than 2-MeOE2 (Newman *et al*, 2004).

2-MeOE2*bis*MATE has therefore been shown to be a potent inhibitor of *in vitro* angiogenesis but, as yet, no studies have been carried out to examine its potential to inhibit angiogenesis *in vivo*. In the present study its ability, and that of 17-Cym-2-MeOE2MATE, to inhibit angiogenesis *in vivo* has been investigated using the Matrigel plug angiogenesis model in C57BL/6J mice. In addition to its anti-angiogenic properties, 2-MeOE2*bis*MATE also inhibits the growth of oestrogen receptor positive (ER+) and negative (ER−) cells (Utsumi *et al*, 2005). It was 10-fold more potent than 2-MeOE2 at inhibiting the proliferation of MCF-7 ER+ breast cancer cells *in vitro* (Newman *et al*, 2004). Its ability to inhibit the growth of xenografts derived from MCF-7 breast cancer cells was therefore also examined in the present study. Tumours obtained from these animals were further examined for evidence that these compounds could inhibit tumour angiogenesis.

## MATERIALS AND METHODS

### Compound and synthesis

**Figure 1:**
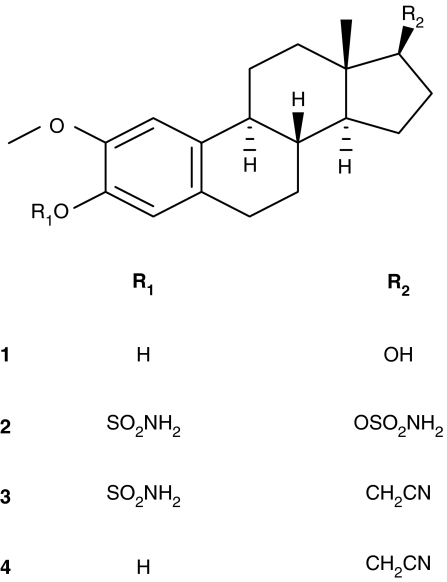
Structure of compounds. Compound **1**, 2-methoxyoestradiol (2-MeOE2); compound **2**, 2-methoxyoestradiol-3,17-*O*-*O*-*bis*-sulphamate (2-MeOE2*bis*MATE); compound **3**, 2-methoxy-3-*O*-sulphamoyl-17*β*-cyanomethyl oestra-1, 3, 5 (10)-triene (17-Cym-2-MeOE2MATE); compound **4**, 2-methoxy-3-hydroxy-17*β*-cyanomethyl oestra-1, 3, 5 (10)-triene (17-Cym-2-MeOE2).

2-MeOE2, 2-MeOE2*bis*MATE, 17-Cym-2-MeOE2MATE and its non-sulphamoylated derivative (2-methoxy-3-hydroxy-17*β*-cyanomethyl oestra-1,3,5 (10)-triene, 17-Cym-2-MeOE2, Figures 1, 4) were synthesised as described previously (Leese *et al*, 2005, 2006; Utsumi *et al*, 2005). All compounds exhibited spectroscopic and analytical data in accordance with their structure and were pure, as shown by high-performance liquid chromatography.

### *In vitro* cell proliferation

The human breast carcinoma cell line MCF-7 (ER+) was obtained from the American Type Culture Collection (LGC Promochem, Teddington, UK) and maintained in Dulbecco's minimal essential medium containing phenol red, supplemented with 10% fetal calf serum and antibiotics (Sigma, Poole, Dorset, UK). Cells were cultured at 37°C under 5% CO_2_ in a humidified incubator.

### Matrigel plug assay

The Matrigel plug assay was a modified version of the methods described previously (Passaniti *et al*, 1992; Prewett *et al*, 1999). Briefly, female C57BL/6J mice (6–8 weeks old) were obtained from Charles River UK Ltd (Margate, Kent, UK). Animals were maintained in positive pressure isolators under a 12 h light–dark cycle and allowed access to food and water *ad libitum*. The experiments were approved by the Imperial College Animal Ethical Review Committee and met the standards required by the UKCCCR guidelines (Workman *et al*, 1998). Mice were anaesthetised, placed on a heated pad (37°C) and injected subcutaneously, into the flanks with 0.5 ml ice-cold Matrigel (Becton Dickinson, Oxford, Oxon, UK) supplemented with 500 ng basic fibroblast growth factor (bFGF; R&D Systems, Oxford, Oxon, UK). Control mice were injected with Matrigel without bFGF. Before the end of each study vascularisation of Matrigel was quantified by injecting mice with FITC-dextran (125 000 molecular weight, Sigma), 0.1 ml of a 0.25 mg ml^−1^ solution intravenously (i.v.), which allowed blood vessels within plugs to be visualised. Animals were killed 20 min after injection, when Matrigel plugs were removed and photographs showing the extent of vascularisation taken using a dissecting microscope (Nikon SMZ1500). Photographs of blood vessels within Matrigel plugs were also obtained using a microscope with a fluorescent light source (Zeiss-Axiovert 200). Quantification of FITC-dextran in the Matrigel plugs was achieved by incubating plugs in 1 ml Dispase reagent (Becton Dickinson) for 16 h at 37°C. The resulting mixture was centrifuged in a microfuge at 13 000 r.p.m. for 30 s. The fluorescence of the resulting supernatants was measured using a fluorimeter (Fluostar plus Optima, BCG, Bucks, UK), excitation at 480 nm, measurement at 520 nm, and quantitated against a standard curve of FITC-dextran (0.4–25 *μ*g ml^−1^).

A preliminary study was carried out to determine the time for optimal neovascularisation of Matrigel plugs to develop. For this plugs were removed from control mice (no added bFGF) at the end of days 7, 10 and 14 and from mice where plugs contained bFGF at the end of days 1, 2, 4, 7, 10 and 14 after Matrigel injection. Having determined the optimal time for neovascularisation, a dose–response study using 2-MeOE2 or 2-MeOE2*bis*MATE was performed in which these compounds were administered daily at 1, 10 or 50 mg day^−1^, *per os*, (p.o.) (in tetrahydrofuran : propylene glycol, THF : PG, 1 : 9, v/v), daily for 7 days. At the end of the study, Matrigel plugs were removed for visualisation and quantification. After identifying doses of 2-MeOE2 and 2-MeOE2*bis*MATE that inhibited vascularisation the number of doses to inhibit neovascularisation (preventative study) or disrupt established vasculature (treatment study) was investigated. For the preventative study mice were dosed with compounds (50 mg kg^−1^, p.o.) for 1–4 days 24 h after Matrigel injection. Seven days after injection of the Matrigel plugs mice were killed and the plugs removed for visualisation and quantification. For the treatment study, neovascularisation was allowed to develop over a 7-day period. Groups of animals were dosed with compounds on day 8 (1-day dosing) or days 8–10 (3 days of dosing) at 50 mg kg^−1^, p.o. The effects on established vasculature were assessed 14 days after the injection of the Matrigel plugs.

A study was also carried out to compare the anti-angiogenic properties of 2-MeOE2*bis*MATE with that of 17-Cym-2-MeOE2MATE and its non-sulphamoylated derivative 17-Cym-2-MeOE2. For this, compounds (20 mg kg^−1^, p.o.) were administered daily for 4 days with plugs being removed for visualisation and quantification on day 8.

### MCF-7 tumour xenograft model

MCF-7 breast cancer cell tumours were established by inoculating cells subcutaneously (10 × 10^6^ cells in 0.1 ml growth factor-reduced Matrigel) into female athymic (*nu/nu*) mice (Harlan, Oxford, Oxon, UK). The growth of these hormone-dependent tumours was stimulated by the implantation of oestradiol-slow release pellets (0.52 mg, 60-day release; Innovative Research of America, Sarasota, FL, USA) 24 h before cell inoculation. When tumours had reached 100 mm^3^ in volume animals were dosed orally with vehicle (THF : PG 1 : 9, v/v), 2-MeOE2 (20 mg kg^−1^), 2-MeOE2*bis*MATE (5 and 20 mg kg^−1^) or 17-Cym-2-MeOE2MATE (5 and 20 mg kg^−1^) daily for 5 days per week for 3 weeks. Tumour volumes were measured weekly with callipers and tumour volumes calculated using the formula *lw*^*2*^*/*2 (where *l* is the length and *w* is the width) with results being expressed as the percentage change in tumour volumes after 1, 2 or 3 weeks of dosing compared with tumour volumes at the start of dosing. At the end of the study, some mice (*n*=3) were injected i.v. with FITC-dextran (0.1 ml of 0.25 mg ml^−1^) 20 min before the animals were killed. Blood vessels in tumours were visualised, photographed and tumour angiogenesis quantified as described previously for Matrigel plugs.

As oestrogen sulphamates are potent inhibitors of steroid sulphatase (STS), its activity was measured in samples of tumour and liver tissues from control and treated animals as described previously (Purohit *et al*, 1995a, 1995b).

### Tumour histology

Tumours derived from MCF-7 cells from mice were immediately frozen and used to prepare cryostat sections (0.6 *μ*m) for immunohistochemical analysis. Sections were stained for Ki67, a marker for cell proliferation, using an anti-Ki67 antibody (AbCam, Cambridge, Cambs, UK) with an ABC complex and chromogen kit which yielded a red stain when positive. The effect of the compounds on angiogenesis was assessed by staining for VEGFR-2 (AbCam), a marker for endothelial cells (Matthews *et al*, 1991; Terman *et al*, 1992). For this, sections were stained using a human VEGFR-2 antibody with a streptavidin peroxidase system with subsequent haemotoxylin counterstaining. Quantification of sections was achieved by subtracting the background staining from the specific staining using image analysis software (Image J, NIH, Bethesda, MD, USA) developed by the NIH that measures the number of stained pixels/field, selected from three random fields from three samples for each treatment group. Isotype control staining was carried out for all specimens.

### Statistics

Data were analysed for statistical significance using Student's *t*-test or analysis of variance (ANOVA).

## RESULTS

### Inhibition of vascularisation in Matrigel plugs

**Figure 2:**
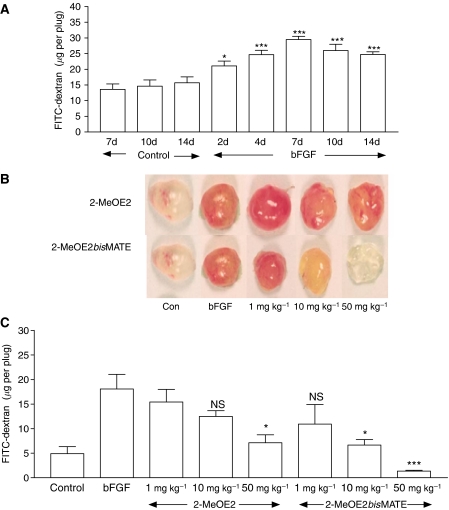
(**A**) Time course for neovascularisation in Matrigel plugs. C57BL/J6 mice were injected subcutaneously with 0.5 ml Matrigel with or without (control) bFGF. Twenty minutes before removing plugs from mice FITC-dextran (0.1 ml of a 0.25 mg ml^−1^ solution) was injected i.v. to facilitate visualisation and quantification of angiogenesis. Plugs were removed on days 2–14 after injection (means±s.e.m., *n*=5, ^*^P<0.05; ^***^P<0.001 compared with corresponding controls). (**B**) Effect of 2-MeOE2 and 2-MeOE2*bis*MATE on Matrigel plug neovascularisation. Compounds were administered orally at the doses indicated for 7 days starting 24 h after Matrigel injection. On day 8 FITC-dextran was injected 20 min before removal of plugs from mice as described above. Representative photographs are shown from five mice per group. (**C**) Quantification of angiogenesis within the Matrigel plugs was achieved after FITC-dextran injection (means±s.e.m., *n*=5, ^*^P<0.05, ^***^P<0.001 compared with the level of vascularisation in Matrigel plugs supplemented with bFGF in animals receiving vehicle only, NS, nonsignificant).

An initial study was carried out to determine the time required for optimal neovascularisation of Matrigel plugs containing bFGF. As shown in Figure 2A, for animals injected with Matrigel lacking bFGF, minimal vascularisation was seen and there was no increase in the level of FITC-dextran fluorescence in plugs retrieved from animals over a 7–14-day period. In contrast, plugs containing bFGF revealed a marked degree of neovascularisation with a maximum increase (116%, *P*<0.001) being achieved by day 7 after plug injection. No further increases were detected on leaving the plugs in animals for up to a further 7-day period. A preliminary study was carried out in which VEGF was added to the Matrigel in addition to bFGF but this did not increase the level of neovascularisation seen above that resulting from the use of bFGF alone (data not shown).

To examine the ability of 2-MeOE2 or 2-MeOE2*bis*MATE to inhibit bFGF-induced plug neovascularisation mice were dosed orally with these compounds for 7 days, starting 24 h after Matrigel injection. Visual inspection of the plug after removal revealed the marked increase in vascularisation, seen as a deep red appearance that occurred in plugs of untreated animals (Figure 2B). For animals dosed with 2-MeOE2 (50 mg kg^−1^) or 2-MeOE2*bis*MATE (10 and 50 mg kg^−1^) there was a marked reduction in the level of neovascularisation. Quantification of angiogenesis revealed modest inhibitory effects of 2-MeOE2 (20 and 43% at 1 and 10 mg kg^−1^ respectively; NS) but a significant (83%, *P*<0.05) reduction at the highest dose tested (Figure 2C). The effect of 2-MeOE2*bis*MATE on neovascularisation was much greater than that of 2-MeOE2. At 10 mg kg^−1^ a similar reduction (87%, *P*<0.05) was detected to that resulting from dosing animals with 50 mg kg^−1^ of 2-MeOE2. At the highest dose of 2-MeOE2*bis*MATE tested the level of angiogenesis in plugs was below that detected in control animals (*P*<0.001). At both 10 and 50 mg kg^−1^ doses, 2MeOE2*bis*MATE significantly inhibited bFGF-induced angiogenesis compared with 2MeOE2.

**Figure 3:**
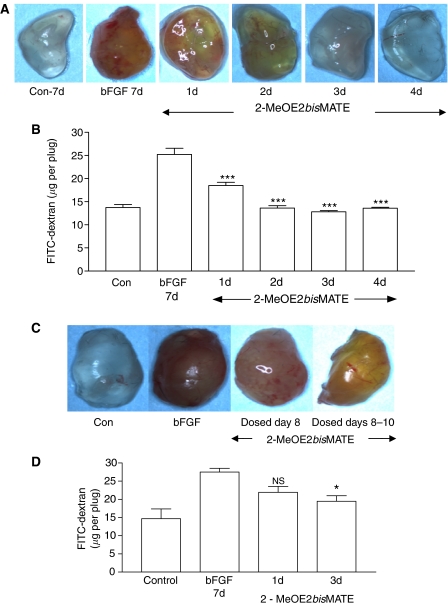
(**A**) The effect of dosing with 2-MeOE2*bis*MATE (50 mg kg^−1^, p.o.) for 1–4 days on neovascularisation of Matrigel plugs. After dosing for 1–4 days plugs were removed on day 7 after Matrigel injection. FITC-dextran was used as described previously to facilitate visualisation and quantification of angiogenesis. Representative photographs of plugs from groups of five animals are shown. (**B**) Quantification of angiogenesis within Matrigel plugs shown in (A) after FITC-dextran injection (means±s.e.m., *n*=5, ^***^P<0.001 compared with bFGF). (**C**) The effect of dosing with 2-MeOE2*bis*MATE (50 mg kg^−1^, p.o.) on established Matrigel plug vasculature. Neovascularisation was allowed to develop for 7 days before animals were dosed for 1 day or daily for 3 days. Plugs were removed at end of day 14 for photography and quantification after FITC-dextran injection. (**D**) Quantification of angiogenesis within Matrigel plugs shown in Figure 3c after FITC-dextran injection (means±s.e.m., *n*=5, ^*^P<0.05 compared with bFGF; NS, nonsignificant).

Having established that a dose of 50 mg kg^−1^ 2-MeOE2*bis*MATE effectively abolished Matrigel plug neovascularisation, this dose was used to investigate the number of doses required to prevent neovascularisation (preventative study) or to disrupt established blood vessels (treatment study), as would be encountered in a therapeutic setting. As shown in Figure 3A and B, 7 days after injection of Matrigel plugs a marked increase in neovascularisation was detected on visual inspection and quantification. Administration of 2-MeOE2*bis*MATE daily for 1, 2, 3 or 4 days, 24 h after Matrigel injection, with removal of plugs 7 days after Matrigel injection, revealed that dosing for 2 days was sufficient to completely blocked plug vascularisation. After letting neovascularisation become established over a 7-day period dosing for 1 day (i.e. day 8), followed by plug removal at end of the study on day 14, resulted in a 44% reduction, with dosing for 3 days (i.e. days 8–10) resulting in a 63% (*P*<0.05) reduction in plug vascularisation (Figure 3C and D).

**Figure 4:**
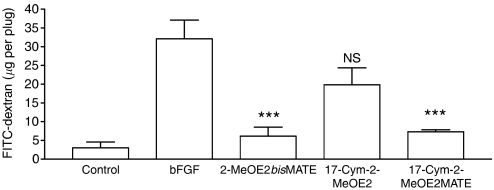
Comparison of the *in vivo* anti-angiogenic efficacy of 2-MeOE2*bis*MATE and 17-Cym-2-MeOE2MATE. Compounds (20 mg kg^−1^) were administered orally, daily for 4 days, 24 h after Matrigel injection, with plug removal on day 7. FITC-dextran was injected 20 min before plug removal after which the level of angiogenesis was quantified (means±s.e.m., *n*=5, ^***^P<0.001, compared with level in plugs supplemented with bFGF for animals receiving vehicle only; NS, nonsignificant).

The efficacy of 2-MeOE2*bis*MATE in the Matrigel plug assay was also compared with that of the cyanomethyl analogues (Figure 4). Both 2-MeOE2*bis*MATE and 17-Cym-2-MeOE2MATE when administered at 20 mg kg^−1^ day^−1^ daily for 4 days, 24 h after Matrigel injection, almost completely inhibited plug vascularisation (89 and 85% respectively, *P*<0.001). In contrast, the non-sulphamoylated analogue, 17-Cym-2MeOE2, did not significantly reduce vascularisation at the dose tested.

### Inhibition of MCF-7 tumour growth and angiogenesis

**Figure 5:**
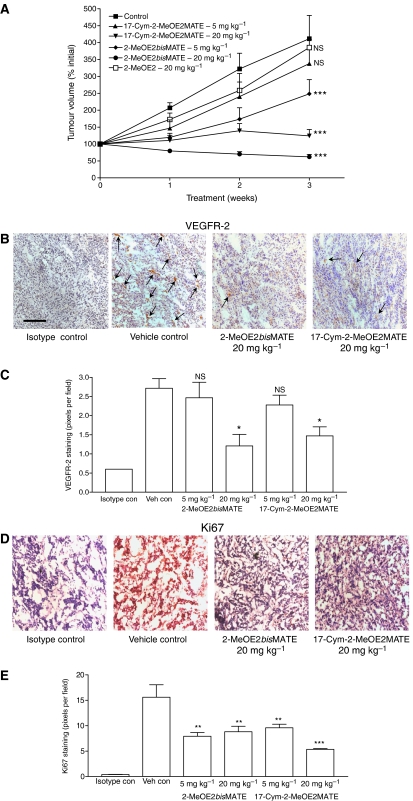
(**A**) Inhibition of growth of tumours derived from MCF-7 cells in nude mice. Animals were dosed orally with 2-MeOE2*bis*MATE or 17-Cym-2-MeOE2MATE (5 mg kg^−1^ or 20 mg kg^−1^) or 2-MeOE2 (20 mg kg^−1^) 5 days per week for 3 weeks. Tumour volumes were measured weekly (means±s.e.m., *n*=5-10, ^***^P<0.001 compared with controls; NS, nonsignificant). (**B**) Section of tumours were prepared and stained for VEGFR-2 (× 200 magnification; scale bar, 1 cm=200 *μ*m)) as indicated by the arrows. (**C**) Quantification of staining for VEGFR-2 revealed that at 20 mg kg^−1^ both 2-MeOE2bisMATE and 17-Cym-2-MeOE2MATE significantly reduced the expression of this receptor (means±s.e.m., *n*=3, ^*^P<0.05, NS, nonsignificant). (**D**) Section of tumours were prepared and stained for Ki67, a marker of cell proliferation (× 200 magnification). (**E**) Quantification revealed that at both doses tested a significant reduction in Ki67 expression occurred (means±s.e.m., *n*=3, ^**^P<0.01, ^***^P<0.001 compared with control).

The ability of 2-MeOE2, 2-MeOE2*bis*MATE and 17-Cym-2-MeOE2MATE to inhibit tumour growth was tested using xenografts derived from oestradiol-stimulated MCF-7 cells (Figure 5A). In the control group, tumour volumes increased by 300% over the 3-week period (Figure 5A). For mice receiving 2-MeOE2 (20 mg kg^−1^, p.o.) the growth of tumours did not differ from that of animals in the control group. In contrast, dosing with 2-MeOE2*bis*MATE resulted in a significant reduction in tumour volumes (52%, *P*<0.001) at the 5 mg kg^−1^ dose with a 38% regression being detected at the 20 mg kg^−1^ dose (*P*<0.001). For 17-Cym-2-MeOE2MATE only the 20 mg kg^−1^ dose was effective, reducing tumour growth by 92% (*P*<0.001) compared with controls. Over the 3-week period for which animals were dosed no significant changes in body weights were detected indicating a lack of toxicity at the doses tested (data not shown). Quantification of tumour angiogenesis by FITC-dextran injection revealed that this decreased over the 3-week period by 44±8% (*P*<0.05) after dosing with 2-MeOE2*bis*MATE and 53±15% (*P*<0.01) on dosing with 17-Cym-2MeOE2MATE. STS activity in livers and tumours was inhibited by >90% by both sulphamoylated compounds (data not shown).

### MCF-7 tumour histology

Histological examination of tumours obtained at the end of the study revealed a high level of staining for the VEGFR-2 in control animals (Figure 5B). Less specific staining was evident in sections prepared from animals treated with 20 mg kg^−1^ of 2-MeOE2*bis*MATE or 17-Cym-2-MeOE2MATE. This was reflected in the quantification of the VEGFR-2 staining, which revealed significant decreases of 72% for 2-MeOE2*bis*MATE and 57% for 17-Cym-2-MeOE2MATE (Figure 5C). At the 5 mg kg^−1^ doses these compounds had minimal effect on VEGRF-2 expression. The decrease in tumour growth and expression of VEGFR-2 resulting from dosing with these compounds was associated with significant decreases in tumour cell proliferation as indicated by the staining and quantification of Ki67 (Figures 5D and E). At the 5 mg kg^−1^ dose both sulphamoylated compounds reduced Ki67 expression (49–68%) in contrast to VEGFR-2, where a reduction in the level of this receptor was only detected at the higher doses of the compounds tested.

### Visualisation of vasculature in Matrigel plugs and MCF-7 tumours

**Figure 6:**
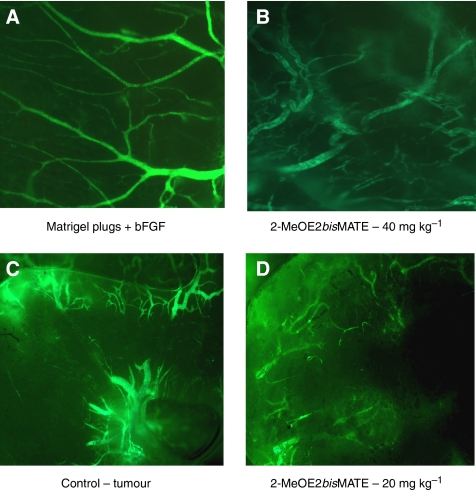
(**A**) Matrigel plugs supplemented with bFGF were removed from mice after FITC-dextran injection. The vasculature was visualised and photographed using a fluorescent microscope. For animals receiving vehicle only the intact vasculature was clearly visible. (**B**) For animals receiving 2-MeOE2*bis*MATE (40 mg kg^−1^, p.o., daily for 4 days) marked disruption of the vasculature occurred. (**C**) Studies with tumours derived from MCF-7 cells in nude mice after FITC-dextran injection revealed a well-established vasculature in mice receiving vehicle. (**D**) For animals receiving 2-MeOE2*bis*MATE (20 mg kg^−1^, p.o.) daily for 21 days disruption of tumour vasculature was detected.

After establishing the FITC-dextran method to visualise Matrigel plug vasculature this technique was further employed to examine the effects of the sulphamoylated compounds on Matrigel plug and tumour vasculature (Figure 6). Matrigel plugs removed from the studies described above were routinely photographed using a fluorescent microscope to visualise the blood vessels. In this particular study, a Matrigel plug assay was performed with 2MeOE2*bis*MATE at 40 mg kg^−1^, p.o. daily for 4 days and Matrigel plugs removed at the end of 7 days. Blood vessels in the control bFGF supplemented plugs had a well-defined branching structure indicative of a functioning capillary network (Figure 6A). In plugs from animals receiving 2-MeOE2*bis*MATE, the fluorescence associated with blood vessels was less intense with evidence of vascular disruption (Figure 6B). In the MCF-7 breast tumour xenograft model study a number of mice were injected with FITC-dextran 20 min before killing (see Materials and Methods section for details). Tumours were removed and the tumour vasculature visualised and photographed using a fluorescent microscope. In MCF-7 tumours obtained from untreated animals, it was possible to see the blood vessel network within the tumours (Figure 6C). In contrast, in tumours from animals treated with 2-MeOE2*bis*MATE (20 mg kg^−1^, p.o. 5/7 days per week for 3 weeks), disruption of tumour vasculature was clearly evident (Figure 6D).

## DISCUSSION

The results from this study demonstrate that oral administration of 2-MeOE2*bis*MATE is able to block angiogenesis *in vivo* in the mouse Matrigel plug model. At the highest dose tested neovascularisation was below the background level detected in Matrigel plugs not containing bFGF demonstrating the potency of this compound as an anti-angiogenic agent. 2-MeOE2*bis*MATE caused significant regression of tumour xenografts derived from MCF-7 breast cancer cells and reduced angiogenesis within these tumours. 17-Cym-2-MeOE2MATE also proved to be a potent inhibitor of angiogenesis in the Matrigel plug model and also significantly reduced the growth of MCF-7 xenografts at the higher dose tested (20 mg kg^−1^). Thus, the 2-methoxy-3-*O*-sulphamoylated oestrogens are emerging as a class of compounds with greatly enhanced *in vivo* efficacy relative to 2-MeOE2. 2-MeOE2 has previously been shown to inhibit angiogenesis *in vivo* in xenografts derived from B16 melanoma cells and MDA-MB-435 ER- breast cancer cells (Fotsis *etal*, 1994; Klauber *et al*, 1997). In addition, the growth of these tumours was also inhibited by oral administration of 2-MeOE2 although relatively high doses (75–100 mg kg^−1^) were required to reduce tumour growth. In the present study, dosing with 2-MeOE2 at 50 mg kg^−1^ confirmed its ability to inhibit angiogenesis in the Matrigel plug model, achieving a similar degree of inhibition to that seen with 2-MeOE2*bis*MATE at 10 mg kg^−1^. This suggests that 2-MeOE2*bis*MATE is at least five times more potent than 2-MeOE2 at inhibiting *in vivo* angiogenesis. This is consistent with results obtained from *in vitro* studies where 2-MeOE2 was less effective at inhibiting the growth of HUVECs or tubule formation in two *in vitro* models of angiogenesis (Newman *et al*, 2004). Our compounds show potent activity at low doses whereas 2MeOE2 at the same low dose is only weakly active. However, higher doses of 2MeOE2 (Huh *et al*, 2006) do produce similar effects to those observed with 2-MeOE2*bis*MATE and 17-Cym-2-MeOE2MATE.

The superior efficacy of 2-MeOE2*bis*MATE and related compounds over that of 2-MeOE2, raises important questions as to why sulphamoylation increases the *in vitro* and *in vivo* potency of this class of compound. It is evident that 2-MeOE2*bis*MATE is not acting as a pro-drug for 2-MeOE2. In a metabolic study in rats, 2-MeOE2 was not detectable in plasma for up to 24 h after the oral or i.v. administration of 2-MeOE2*bis*MATE (Ireson *et al*, 2004). It is now known that sulphamoylated derivatives of oestrogens have a superior pharmacokinetic profile and are more resistant to metabolism than their non-sulphamoylated counterparts (Newman *et al*, 2006). Oestrogen sulphamates, such as oestrone-3-*O*-sulphamate (EMATE) were originally identified as potent STS inhibitors, a property shared with 2-MeOE2*bis*MATE (Raobaikady *et al*, 2005). EMATE was not developed for breast cancer therapy as it became evident that oestrogen sulphamates had enhanced oestrogenicity when administered orally to rats (Elger *et al*, 1995). In a uterine weight gain assay, oestradiol sulphamate proved to be five times more active than ethinyloestradiol and 100 times more active than oestradiol, on oral application. The reasons for the enhanced oestrogenicity associated with oestrogen sulphamates is now known to result from their ability to be taken up into erythrocytes where they bind to carbonic anhydrase II (Ho *etal*, 2003; Vicker *et al*, 2003). They transit the liver in the erythrocytes, and are thus protected from first-pass inactivation, with subsequent release into the systemic circulation. Support for such a protective role for sulphamoylation was also obtained in the present study in which the ability of 17-Cym-2-MeOE2MATE and its non-sulphamoylated analogue were tested for their ability to inhibit *in vivo* angiogenesis. Both of these compounds were effective at inhibiting HUVEC growth and the formation of microtubules in *in vitro* models of angiogenesis (unpublished data). This contrasts with the results obtained in the present study which demonstrated that while 17-Cym-2-MeOE2MATE was equipotent with 2-MeOE2*bis*MATE at inhibiting Matrigel plug vascularisation the non-sulphamoylated analogue, 17-Cym-2-MeOE2, was much less effective at the same dose level. Thus, the sulphamoylation of oestrogens having a 2-methoxy function is an important mechanism for enhancing the potency and oral bioavailability of this class of compound. In contrast to EMATE, 2-MeOE2*bis*MATE and other compounds tested at 2000-fold higher dose than oestrone are devoid of oestrogenic properties in ovariectomised rats (data not shown) indicating lack of binding to ER.

In addition to inhibiting the neovascularisation of Matrigel plugs both 2-MeOE2*bis*MATE and 17-Cym-2-MeOE2MATE proved effective at inhibiting the growth of tumours derived from MCF-7 cells in nude mice. At 20 mg kg^−1^, 2-MeOE2 did not inhibit the growth of these tumours, a finding in keeping with results from *in vitro* growth studies where 2-MeOE2*bis*MATE proved 10 times more active than 2-MeOE2 (Newman *et al*, 2004). The reduction in tumour growth was consistent with the 51–68% decrease in the expression of Ki67. At 20 mg kg^−1^ 2-MeOE2 had no effect on Ki67 expression (data not shown). At the doses tested, that is up to 20 mg kg^−1^ over a 3-week period, 2-MeOE2*bis*MATE and 17-Cym-2-MeOE2 appeared to be devoid of any toxic effects as no significant effects on animal weight was detected.

2-MeOE2*bis*MATE is thought to act like 2-MeOE2, by binding to the colchicine-binding site on tubulin and altering microtubule stability (MacCarthy-Morrogh *et al*, 2000; Raobaikady *et al*, 2005). It induces Bcl-2 phosphorylation and apoptosis in endothelial and cancer epithelial cells. It was recently reported that 2-MeOE2, by disrupting microtubule stability, causes downregulation of the pro-angiogenic transcription factor Hif-1*α* in PC3 and MDA-MB-231 cancer cells with a subsequent reduction in VEGF production (Mabjeesh *et al*, 2003). While VEGF levels were not measured in the present study, 2-MeOE2*bis*MATE and 17-Cym-2-MeOE2MATE were found to cause a significant reduction in the levels of one of the VEGF receptors, VEGFR-2, however this is probably caused by these compounds ability to reduce angiogenesis.

Additional experiments were carried out to examine if this class of angiogenesis inhibitors only blocks the formation of new blood vessels or whether they can also disrupt existing vasculature. For this, animals were tested in a ‘preventative’ or ‘treatment’ setting as described previously. Results from these studies clearly demonstrate that 2-MeOE2*bis*MATE is able to almost completely block neovascularisation of Matrigel plugs when given for 2–4 days after Matrigel injection. After allowing neovascularisation to develop for 7 days, dosing for 1 or 3 days, with quantification at end of day 14, revealed that 2-MeOE2*bis*MATE could significantly reduce established vasculature, although to a lower degree than when given in the preventative setting. Inspection of blood vessel structure in Matrigel plugs and tumours revealed gross disruptions of the vasculature. As tumours for these studies were obtained from mice after dosing for a 3-week period these results suggest that 2-MeOE2*bis*MATE will be able to target established vasculature in addition to inhibiting the neovascularisation process.

In summary, 2-MeOE2*bis*MATE and 17-Cym-2-MeOE2MATE have been shown to be potent inhibitors of *in vivo* angiogenesis in the mouse Matrigel plug assay. In addition, both compounds were able to suppress the growth of xenografts derived from oestradiol-stimulated MCF-7 cells in nude mice. Thus, these compounds have a dual anti-proliferative, anti-angiogenic action that should enhance their therapeutic efficacy. These compounds were at least 10 times more active than 2-MeOE2 at inhibiting the neovascularisation of Matrigel plugs indicating that sulphamoylation confers significant additional potency to this class of compound. In view of the potent anti-angiogenic and tumour growth inhibitory properties of this class of compounds, they should have considerable therapeutic potential for the treatment of a wide range of cancers.
